# Aided Phytoremediation to Clean Up Dioxins/Furans-Aged Contaminated Soil: Correlation between Microbial Communities and Pollutant Dissipation

**DOI:** 10.3390/microorganisms7110523

**Published:** 2019-11-03

**Authors:** Hacène Meglouli, Joël Fontaine, Anthony Verdin, Maryline Magnin-Robert, Benoît Tisserant, Mohamed Hijri, Anissa Lounès-Hadj Sahraoui

**Affiliations:** 1 Université du Littoral Côte d’Opale, UCEIV-EA 4492, SFR Condorcet FR CNRS 3417, CS 80699, F-62228 Calais CEDEX, France; 2 Institut de Recherche en Biologie Végétale, Université de Montréal and Jardin botanique de Montréal, 4101 Rue Sherbrooke Est, Montréal, QC H1X 2B2, Canada; 3 AgroBioSciences, Mohammed VI Polytechnic University, Lot 660, Hay Moulay Rachid, Ben Guerir 43150, Morocco

**Keywords:** phytoremediation, dioxins/furans, microbial community, arbuscular mycorrhizal fungi, biosurfactant

## Abstract

To restore and clean up polluted soils, aided phytoremediation was found to be an effective, eco-friendly, and feasible approach in the case of many organic pollutants. However, little is known about its potential efficiency regarding polychlorinated dibenzo-p-dioxins and furans-contaminated soils. Thus, phytoremediation of aged dioxins/furans-contaminated soil was carried out through microcosm experiments vegetated with alfalfa combined with different amendments: an arbuscular mycorrhizal fungal inoculum (*Funneliformis mosseae*), a biosurfactant (rhamnolipids), a dioxins/furans degrading-bacterium (*Sphingomonas wittichii* RW1), and native microbiota. The total dioxins/furans dissipation was estimated to 23%, which corresponds to 48 ng.kg^−1^ of soil, after six months of culture in the vegetated soil combined with the four amendments compared to the non-vegetated soil. Our findings showed that the dioxins/furans dissipation resulted from the stimulation of soil microbial enzyme activities (fluorescein diacetate hydrolase and dehydrogenase) and the increase of bacterial abundance, richness, and diversity, as well as fungal diversity. Amplicon sequencing using Illumina MiSeq analysis led to identification of several bacterial (Bacillaceae, Sphingomonadaceae) and fungal (*Chaetomium*) groups known to be involved in dioxins/furans degradation. Furthermore, concomitant cytotoxicity and dioxins/furans concentration decreases were pointed out in the phytoremediated soil. The current study demonstrated the usefulness of combining different types of amendments to improve phytoremediation efficacy of aged dioxins/furans-contaminated soils.

## 1. Introduction

Polychlorinated dibenzo-p-dioxins (PCDD) and polychlorinated dibenzo-furans (PCDF) are persistent chlorinated organic compounds, ranked among the priority environmental contaminants in the world [1]. These PCDD/F are produced from different anthropogenic activities such as combustion, metal processing, and chemical manufacturing [2]. PCDD/F composed of two aromatic rings linked via one (PCDF) or two atoms of oxygen (PCDD) and one to eight related chlorine atoms, are very stable and extremely hydrophobic halogenated chemicals [3]. They are found in the environment, including air, water, and soil. Due to their persistence in the environment, they tend to contaminate the food chain, with accumulation in dairy food, meat, and fish, and then pose serious risks to human health [4,5]. Moreover, humans may also become exposed to PCDD/F by inhalation or dermal contact [6]. That is why their elimination from polluted soils has become urgent. Among remediation methods, phytoremediation has been found to be an eco-friendly and feasible approach in the case of many organic pollutants such as pesticides, petroleum hydrocarbons, and chlorinated compounds [7]. However, little is known about its efficiency regarding PCDD/F-contaminated soils. So far, only the study of Wang and Oyaizu [8] showed the ability of white clover to remove dibenzofuran after two months of culture. However, a variety of plant species has been tested and successfully used for the remediation of other organic contaminants. For example, alfalfa can efficiently decontaminate polluted soil with polychlorinated biphenyls due to its extensive root system as well as its high adaptability and tolerance to survive in stressed environments [9,10,11]. Generally, plants can promote the degradation of chlorinated organic pollutants in the soil thanks to the root exudates, which stimulate microbial metabolism in the rhizosphere [12,13]. However, phytoremediation efficiency is often limited because of the pollutant phytotoxicity and/or the low pollutant bioavailability to the rhizosphere microbial populations in regard to the high adsorption of the PCDD/F on soil organic matter [14,15]. To enhance the bioavailability of hydrophobic organic compounds in soil, various solubilizer agents such as biosurfactants have been used in phytoremediation experiments [16,17,18]. In particular, some studies have shown that environmentally friendly biosurfactants such as rhamnolipids have the ability to effectively solubilize and mobilize organic compounds adsorbed on soil particles [19]. These amphiphilic compounds have many advantages over synthetic surfactants in remediation for contaminated soils, such as non-toxicity and high biodegradability [20]. On the other hand, it was shown that arbuscular mycorrhizal fungi (AMF) could also improve phytoremediation effectiveness by enhancing pollutant dissipation and by providing protection to the plants against the pollutant toxicity [21,22]. Some investigations showed that AMF can contribute to increase the biodegradation of chlorinated pollutants such as polychlorinated biphenyls in the plant rhizosphere [23,24]. Finally, another way to enhance phytoremediation efficacy relies on the improvement of the degradative capacity of contaminated soil through the introduction of a specific strain or microbial consortia [25]. This strategy has proven to be successful in cleaning up hydrocarbon-polluted soil [24] but no data are available regarding its efficiency in PCDD/F-contaminated soil bioremediation. 

Although the potential role in phytoremediation of biosurfactants and/or AMF or bacteria with degrading capacity is largely described in the literature, these different amendments were rarely combined in the same experiment. On the other hand, little is known about the rhizosphere microbial community structure of PCDD/F-contaminated soils. This knowledge is important to extend our understanding on ecological implication of the complex interaction between plant-rhizosphere microbiome and the dissipation of pollutants in soil. Thus, a microcosm experiment using alfalfa (*Medicago sativa* L.) aided with a combination of several biological amendments (an AMF inoculum, rhamnolipids, a dioxin-degrading bacteria *S. wittichii* RW1, and the native telluric microbiota) was carried out during six months of culture. The main objectives of the present work were to study (1) the potential PCDD/F dissipation by aided phytoremediation, (2) the structures of bacterial and fungal communities in PCDD/F contaminated rhizosphere using 16S rRNA gene and ITS1 region amplicon sequencing with Illumina MiSeq, respectively, (3) and the contaminated soil toxicity before and after application of aided phytoremediation.

## 2. Materials and Methods 

### 2.1. Experimental Design and Setup

In December 2013, soil samples were collected at 0–30 depth from a PCDD/F-contaminated site, which is located at Halluin in the northern France (50°45′53″ N, 3°7′43″ E), near an old incineration plant in activity for 30 years. Soil samples were dried, homogenized and sieved through a 2 mm sieve, and then sterilized (45 kGy, gamma ray) (Ionisos company, Sablé-sur-Sarthe, France) to inactivate the native AMF and to prevent spontaneous mycorrhizal colonization. The physico-chemical properties of the sterilized soil are presented in Table 1. The PCDD/F content was artificially increased by spiking the soil with an organic extract to increase the bioavailable fraction of PCDD/F. The pollutant extract from the contaminated sterilized soil of Halluin was prepared with toluene/ethanol mixture (10/1) using the microwave (Multiwave 3000, Anton Paar, Les Ulis, France) at 50 °C, and at 20 bars PCDD/F were taken back in acetone and introduced into the contaminated Halluin soil. Only 1/10 of the final soil weight was doped. The doped soil was then left overnight under a fume cupboard to evaporate the acetone, and then mixed with the undoped soil [26]. This doping allowed us to increase the PCDD/F concentration of the aged-contaminated soil by approximately 25% and to obtain the final concentration of 260 ng kg^−1^ corresponding to 26 ng TEQ kg^−1^. From this sterilized and doped soil, six conditions were designed: Sterile soil only (non-vegetated microcosms, control), non-inoculated alfalfa (A), alfalfa combined with *F. mosseae* (A+Fm), plus *S. wittichii* RW1 (A+Fm+RW1), plus rhamnolipids Rh (A+Fm+RW1+Rh), plus microbiota µb (A+Fm+RW1+Rh+µb) grown in microcosms (400 mL, diameter 7.5 cm) (Table 2). 

For all vegetated conditions (A+Fm; A+Fm+RW1; A+Fm+RW1+Rh; A+Fm+RW1+Rh+µb), microcosms were sown with 10 pre-germinated seeds of alfalfa. For AMF non-inoculated conditions (A and non-vegetated), each microcosm containing 270 g of the polluted soil were mixed with 30 g of autoclaved commercial AMF inoculum (two times at 121 °C during 30 min). For the alfalfa combined with *F. mosseae* condition (A+Fm, A+Fm+RW1; A+Fm+RW1+Rh; A+Fm+RW1+Rh+µb), microcosms were filled with 270 g of the polluted soil and 30 g of commercial AMF (10 propagules/g) consisting of *F. mosseae* (Fm, MycAgro Ltd., Bretenière, France). 

For A+Fm+RW1, A+Fm+RW1+Rh, and A+Fm+RW1+Rh+µb conditions, the bacterial strain kindly provided by Erica Hartmann of the CEA laboratory of Marcoule (France) was isolated from an agar medium on a Petri dish and then cultured in a liquid LB medium for 48 h with agitation (50 rpm) at 37 °C. After centrifugation at 1000 rpm for 5 min, the bacterial pellet was taken up in a Bushnell–Haas solution. After 4 days of culture, each microcosm received a volume of 5 mL of the bacterial solution at optical density of 0.5 absorbance units (*λ* = 560 nm). For A+Fm+RW1+Rh and A+Fm+RW1+Rh+µb conditions, a rhamnolipids were produced by fermentation/separation/purification process using *Pseudomonas aeruginosa* in canola oil substrate and contained a mixture of both mono- and di-rhamnolipids with a usual 3:2 ratio, respectively. Their chemical names are acid, 3-((6-deoxy-2-*O*-(6-deoxy-alpha-l-mannopyranosyl)-alpha-Lmannopyranosyl) oxy), 1-(carboxymethyl)octyl ester, mixt. with 1-(carboxymethyl) octyl 3-((6-deoxy-alpha-l-mannopyranosyl) oxy) decanoate (AGAE Technologies, Corvallis, OR, USA). Further, 40 mL of rhamnolipids solution (0.1%) were added after 1 week of culture in the microcosms to obtain a final concentration of 100 mg kg^−1^ of soil. That is, concentrations 9–10 times higher than critical micelle concentration. The recommended concentration levels (Wt Vol^−1^) by AGAE supplier is 0.001%–5%. Finally, the native microbiota without AMF was re-introduced to the A+Fm+RW1+Rh+µb condition by adding 10 mL in each microcosm of non-sterile polluted soil suspension filtered at 20 µm. The experiment was set up with six treatments × three repetitions per treatments × ten plants in each microcosm for six months of culture in the semi-controlled conditions with room temperature, light (12 h/12 h). The plants were watered every two days and randomized every seven days.

### 2.2. Arbuscular Mycorrhizal Root Colonization 

Arbuscular mycorrhizal root colonization was visualized after 6 months of culture by microscopic observation (Olympus BX40F, Tokyo, Japan). Fresh roots were sampled and stained with trypan blue according to the method reported in Mustafa et al. [27]. The root colonization rate was calculated using the grid line intersect method [28]. After that, three slides in each microcosm and 15 fragments per slide were made. In total, 135 segments of 1 cm from three different microcosms were taken for observation under an optical microscope (×100 magnification). Three intersections per root fragment were examined. In total, 405 observations per condition were analyzed to estimate the total, arbuscular, and vesicular mycorrhizal colonization.

### 2.3. Determination of Plant Growth 

After six months of culture, alfalfa roots and shoots were collected to estimate the plant growth under various conditions. Roots and shoots were rinsed with sterile water, lyophilized during 48 h, and weighed to determine their dry weights. Thirty plants were used as biological replicates per condition. 

### 2.4. Estimation of Pigment Contents

Chlorophyll a, chlorophyll b, and carotenoids were extracted from 25 mg of fresh frozen composite leaves of 10 plants stored at −80 °C according to the method reported in Magnin-Robert et al. [29]. Pigment concentrations were estimated by spectrophotometry based on the absorbance coefficients determined by Lichtenthaler [30]. Results were expressed in micrograms of pigments per milligrams of fresh weight (FW).

### 2.5. Extraction, Estimation of Soil PCDD/F Toxic Equivalent Values

Toxicity levels of PCDD/F have been determined by gas chromatography/high resolution mass spectrometry (GC/HRMS) analysis for congener concentrations and reported as toxic equivalent values (TEQ) as previously described by Meglouli et al. [31]. The derived toxic equivalents (TEQs) can be used for hazard and risk assessment of halogenated aromatic mixtures. It is the sum of each congener multiplied by the toxic equivalency factor for that congeners [32]. 

### 2.6. Soil Enzyme Assays 

#### 2.6.1. Soil Dehydrogenase Activity 

The measure of rhizosphere dehydrogenase activity was based on the determination of triphenyl formazan formed from the reduction of 2,3,5-triphenyl tetrazolium chloride (TTC) in the soil according to a modified Tabatabai method [33]. Three replicate samples of 6 g moist soil were mixed with 0.06 g CaCO_3_, 1 mL of a 3% (w v^−1^) TTC, and 2.5 mL distilled water. After 24 h of incubation at 37 °C, 10 mL of methanol was added. The tubes were then incubated for 1 h under horizontal agitation (200 rpm). This last step was repeated three times. After centrifugation at 2000 rpm for 5 min, absorbance of the supernatant was read spectrophotometrically at 485 nm. Dehydrogenase activities in the samples were calculated by using calibration graphs. Results are expressed as µg g^−1^ of soil 24 h^−1^.

#### 2.6.2. Soil Fluorescein Diacetate Hydrolase 

One gram of moist soil was added to 7.5 mL of 60 mM phosphate buffer and 100 μL of a 1 M fluorescein di-acetate substrate (Sigma-Aldrich, Saint-Quentin-Fallavier, France) according to the modified Adam and Duncan method [34]. After incubation at 30 °C with agitation (50 rpm) for 1 h, the reaction was stopped by adding 1 mL of acetone to 1 mL of supernatant. After centrifugation (10,000 rpm for 5 min), 200 μL of supernatant were deposited in a 96-well microplate. The fluorescence of the product (fluorescein) was read using a spectrofluorimeter (Fluoroskan Ascent, Thermo Scientific, Illkirch, France) (emission 523 nm, excitation 494 nm). The standard range was carried out using fluorescein solution (Sigma-Aldrich, Saint-Quentin-Fallavier, France) at 100 μg mL^−1^. Results are presented in µg g^−1^ of soil.

### 2.7. Cytotoxicity Tests

The evaluation of the overall cytotoxicity of soil was conducted before and after the phytoremediation using the Fm+RW1+Rh+µb condition because this condition gives the most important PCDD/F reduction. It consisted of exposing two human cell lines to organic extracts of 160 g of contaminated soil by PCDD/F before and after phytoremediation using toluene as an extraction solvent. The two cell lines that were used were: (1) BEAS-2B lung cells originally derived from normal bronchial epithelial human cells (European Collection Authenticated Cell Cultures ECACC, Wiltshire, UK; reference: 95102433), and (2) HepG2 hepatic cells derived from hepatocellular carcinoma human cells (American Type Culture Collection Company). After 48 h of exposure of 200 µL of PCDD/F extract, possible toxicity was evaluated by means of two complementary enzymatic activities on the both cell lines. The lactate dehydrogenase makes it possible to demonstrate the increase of the membrane permeability via the determination of the cytosolic enzyme, which could be released in the cell culture medium after alteration of the membrane (Cytotoxicity Detection Kit lactate dehydrogenase, Roche Diagnostics, Meylan, France), and the mitochondrial dehydrogenase is used to measure mitochondrial metabolic activity dysfunction (Cell Proliferation Reagent WST-1, Roche Diagnostics). Cytotoxicity tests were performed in 96-well culture microplate with 2 × 10^4^ cells/200 µL culture medium per well. In addition to the various conditions tested, eight replicates corresponding to the control (unexposed cells) and eight wells corresponding to the positive control (100% of cell death) were carried out, to which Triton X-100 (2% v v^−1^) was added before performing the assay. Results are presented by the decrease percentage of lactate dehydrogenase and increase percentage of mitochondrial dehydrogenase versus control soil before phytoremediation condition.

### 2.8. Soil DNA Extraction and DNA Quantification

From 250 mg of control non-vegetated and A+Fm+RW1+Rh+µb soils, genomic DNA was isolated using the NucleoSpin^®^ Soil DNA isolation kit (Macherey-Nagel, Düren, Germany), according to manufacturer instructions. The DNA quality from different samples were resolved by gel electrophoresis on 1% (w v^−1^) agarose gels, and images of gels stained with Ethidium bromide (Molecular Probes, Eugene, USA) were captured with Gel Doc (BioRad, Hercules, CA, USA). All DNA extracts were diluted to 0.5 ng μL^−1^ for subsequent analyses. The Quant-iT™ dsDNA High-Sensitivity Assay Kit (Invitrogen, Carlsbad, CA, USA) was used to quantify the extracted DNA concentration. The protocol of High Sensitivity Quant-iT™ kit (Molecular Probes, Invitrogen, Paisley, UK) was used to determinate DNA concentrations of each samples. The StepOne™ Real-Time PCR System (excitation 485 nm, emission 517 nm) was used to measure the fluorescence. The concentration of all samples was calculated using a standard curve with Sigma Plot.

### 2.9. PCR and Amplicon Sequencing

PCR amplification of 16S rDNA of Bacteria and Archaea were performed using, respectively, primer pairs 341F (5′-CCTACGGGNGGCWGCAG-3′) and 805R (5′-GACTACHVGGGTATCTAATCC-3′) [35], ARCH517F (5′-GCYTAAAGSRNCCGTAGC-3′) and ARCH909R (5′-TTTCAGYCTTGCGRCCGTAC-3′) [36], and fungal ITS regions was performed by using ITS1F (5′-CTTGGTCATTTAGAGGAAGTAA) and 58A2R (5′-CTGCGTTCTTCATCGAT-3′) [37,38]. PCR amplifications were performed once on each DNA sample, totaling 18 PCR reactions of soil samples (2 conditions × 3 replicates × 3 taxa: Bacteria, Fungi and Archaea). Adapters CS1: ACACTGACGACATGGTTCTACA and CS2: TACGGTAGCAGAGACTTGGTCT were added to each forward and reverse primers, respectively. Three pairs of primers were used, as follows: 341F/805R for bacterial 16S rDNA, ITS1F/58A2R for fungal ITS, and ARCH517F/ARCH909R for archaeal 16S rDNA. A unique multiplex identifier (FLD) tag was added to each PCR reaction. A total of three libraries were constructed for Bacteria, Archaea, and Fungi (one library per taxon). Each library was then generated by pooling equal amount of amplified DNA, followed by a cleaning step of each pool with a ratio of 0.85 of AMPure beads (Beckman Coulter). Libraries were quantified using the Kapa Illumina GA with Revised Primers-SYBR Fast Universal kit (Kapa Biosystems, Wilmington, MA,). Average size fragment is determined using a LabChip GX (PerkinElmer, Waltham, MA, USA) instrument. PCR reactions were performed in a final volume of 25 µL containing 0.2 mM of dNTP, 0.6 µM of each primer, 1.25 µL of Dimethyl sulfoxide (DMSO), 1 ng of DNA, and 0.5U of *Taq* DNA polymerase (Roche, Mississauga, ON, Canada). PCR were run in a thermocycler using the following parameters: Initial denaturation at 94 °C for 2 min, 30 cycles of 94 °C for 30 s, 55 °C for 30 s, 72 °C for 30 s, and a final elongation at 72 °C for 7 min. PCR reaction, Barcode multiplexing, and Illumina MiSeq were performed at the Genome Quebec Innovation Center at McGill University (Montreal, QC, Canada).

### 2.10. Bioinformatic Analysis

Brazilian Microbiome Project with some modifications (http://www.brmicrobiome.org/16sprofiling-pipeline-illumina) was used to process the Illumina datasets. The sequences of the partial 16S rRNA gene for bacteria and ITS for fungi were processed in Mothur (v.1.34.4) [39] and Quantitative Insights Into Microbial Ecology (QIIME) (v.1.9.1) [40]. Mothur was used in the first part of the pipeline, and this part was composed of five steps: i) Merge the forward and reverse sequences reads within the *make.contigs* command; ii) trim off primers with *trim.seqs* command; iii) *trim.seqs* command was used with the options pdiff = 2, maxambig = 0, qwindowsize = 50 to trim the sequences; iv) we discarded singletons using the “unique.seqs” and “split.abund” commands with a cutoff of 1; and v) finally, the individual files were obtained after the separation of merged. The next part was performed in QIIME. The gold.fa database (http://drive5.com/uchime/gold.fa) was used to check chimera and the sequences were clustered into operational taxonomic units (OTU) according to their sequence similarity (97%) using the USEARCH v8.1. Reads were first clustered against the Greengenes_13_8 database for bacteria and archaea) (http://greengenes.secondgenome.com/) and the UNITE database (https://unite.ut.ee/) for fungi. Taxonomy was assigned to the representative sequence of each OTU using uclust. Datasets were randomly standardized by subsampling to the sample size that showed the lowest number of reads. For bacteria, we initially retrieved 91,236 reads and retained 36,279 (6046 per sample) after quality filtering in QIIME and Mothur. For fungi, initially, we received 43,194 reads and retained 17,770 reads (2962 reads per samples). Finally, for archaea we received 223,218 reads and retained 79,299 reads (13,216 per sample) after filtering (Table S1). The relative abundances were calculated with Excel on the different datasets to visualize the percentage of the taxonomic affiliations across AMF inoculation and plant species. 

### 2.11. Statistical Analysis 

All statistical analyses were performed using XLSTAT 19.1. The Shapiro and Bartlett tests were used to verify the normality and homoscedasticity of the data, respectively. Data were analyzed statistically by means comparison of 3 replicates by the ANOVA one-way, Mann–Whitney, and Kruskal–Wallis tests (with Bonferroni correction of the *p*-value). Different letters following values indicate statistically significant differences between various conditions. Data of mycorrhizal colonization were converted to arcsine values before ANOVA one-way test. Standard deviations were calculated by Microsoft Office Excel 2007 for all data.

## 3. Results

### 3.1. Mycorrhizal Root Colonization and Root Growth of Alfalfa 

Whereas no AMF structures were observed in the absence of inoculation, mycorrhizal hyphae, arbuscules, and vesicles were clearly observed in roots of alfalfa inoculated with *F. mosseae*. Total mycorrhizal colonization ranged from 30 to 47%, arbuscules from 7 to 12%, and vesicles from 19 to 31% (Table 3). The lower total mycorrhizal and vesicular colonizations were observed in the A+Fm+RW1+Rh+µb condition, while the higher total mycorrhizal, arbuscular, and vesicular colonizations were obtained in alfalfa roots added only with Fm (A+Fm) (Table 3). In parallel to the mycorrhizal colonization, a lower root weight of alfalfa with the A+Fm+RW1+Rh condition was observed (Table 3). In detail, the means of root dry weight in different conditions varied from 39 to 76 mg plant^−1^ (Table 3). 

### 3.2. Shoot Growth and Photosynthetic Pigment Content of Alfalfa 

**Figure 1:**
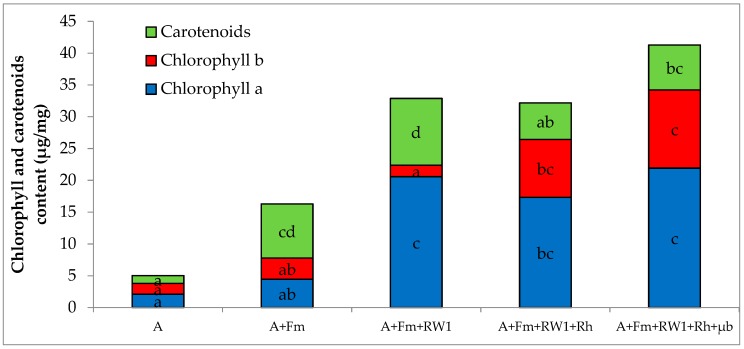
Chlorophyll a and b and carotenoids content in leaves of non-inoculated alfalfa (A), combined with *F. mosseae* (A+Fm), plus *S. wittichii* RW1 (A+Fm+RW1), plus rhamnolipids Rh (A+Fm+RW1+Rh), plus microbiota µb (A+Fm+RW1+Rh+µb) grown in microcosms for six months on the aged polluted soil. Different letters indicate significant differences between conditions according to the Kruskal–Wallis test (*p* ≤ 0.05). Values are means ± s.d. (*n* = 5).

The shoot dry weight of alfalfa cultivated on the historical PCDD/F polluted soil (A) reached 97 mg plant^−1^ after six months. Significant improvements of the shoot weight were observed with all of the conditions, by 1.8-fold with A+Fm+RW1, 1.9-fold with A+Fm+RW1+Rh, 2.1-fold with A+Fm+RW1+Rh+µb, and 2.9-fold with A+Fm (Table 3). The photosynthetic pigment contents in the leaves of alfalfa grown alone (A) were estimated to about 1.5 µg mg^−1^ for each pigment (Figure 1). 

For the other conditions, significant increases of the three pigment contents were observed except for chlorophyll b in A+Fm and A+Fm+RW1 conditions. In details, for chlorophyll a, significant increases of 2.1, 8.2, 9.7, and 10.4 times were observed in A+Fm, A+Fm+RW1+Rh, A+Fm+RW1, and A+Fm+RW1+Rh+µb, respectively. For chlorophyll b, significant increases of 5.4 and 7.3 times were observed in A+Fm+RW1+Rh and A+Fm+RW1+Rh+µb, respectively. For carotenoids, significant increases of 4.7, 5.8, 6.9, and 8.6 times were observed in A+Fm+RW1+Rh, A+Fm+RW1+Rh+µb, A+Fm, and A+Fm+RW1, respectively.

### 3.3. Total PCDD/F Dissipation 

**Figure 2:**
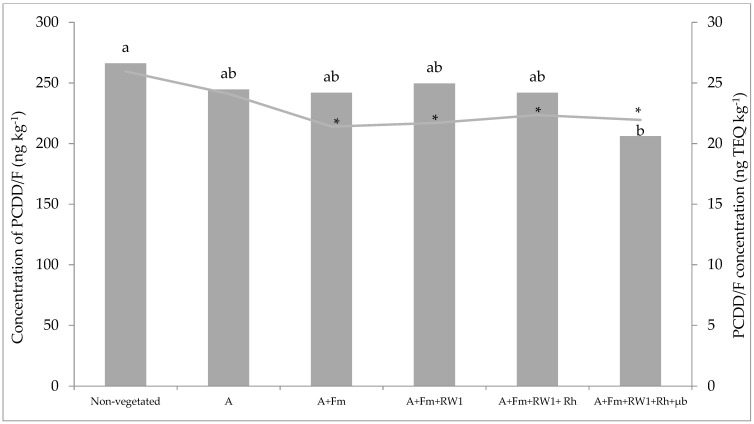
Residual polychlorinated dibenzo-p-dioxins/furans (PCDD/F) concentrations in the polluted soil after six months of culture in ng.kg^−1^ (histogram) and ng TEQ kg^−1^ (curve) for the different conditions. Conditions consisted of non-vegetated soil, non-inoculated alfalfa (A), combined with *F. mosseae* (A+Fm), plus *S. wittichii* RW1 (A+Fm+RW1), plus rhamnolipids Rh (A+Fm+RW1+Rh), plus microbiota µb (A+Fm+RW1+Rh+µb) grown in microcosms for six months. Different letters indicate significant differences between conditions according to the Kruskal–Wallis test (*p* ≤ 0.05). (*) indicate significant differences between conditions compared to the control non-vegetated soil. Values are means ± s.d. (*n* = 3).

After six months of culture, total toxic equivalent PCDD/F concentrations in non-vegetated soils was about 26 TEQ ng kg^−1^. Significant decreases, ranging from 18 to 21.3%, were observed in toxic equivalent PCDD/F concentrations in A+Fm, A+Fm+RW1, A+Fm+RW1+Rh, and A+Fm+RW1+Rh+µb conditions compared to non-vegetated soil (Figure 2). On the other hand, only the A+Fm+RW1+Rh+µb condition showed significant difference compared to non-vegetated soil in raw values. In this condition, total PCDD/F dissipation was estimated to be 23% corresponding to 48 ng kg^−1^ (5.47 TEQ ng kg^−1^) compared to non-vegetated soil. Moreover, the toxic equivalent PCDD/F accumulation in shoots and roots were estimated, respectively, to 0.01 and 0.24% after six months of culture in the A+Fm+RW1+Rh+µb condition (Figure S1, Supplementary materials). 

### 3.4. Estimation of the Polluted Soil Cytotoxicity after phytoremediation with A+Fm+RW1+Rh+µb Condition

Regarding lung cells (Beas2B), lactate dehydrogenase activity was significantly decreased (*p* < 0.0001) by 44% compared to the soil before phytoremediation, which means an important decrease in soil cytotoxicity (Table 4). However, no significant difference in mitochondrial dehydrogenase activities was observed in A+Fm+RW1+Rh+µb and soil sample before phytoremediation. In liver cells (HepG2), mitochondrial dehydrogenase activity increased significantly (*p* < 0.0001) by 18% compared to control soil before phytoremediation, which means a decrease in soil cytotoxicity, and lactate dehydrogenase activity was significantly decreased (*p* = 0.0001) by 5% (Table 4). These results clearly showed that the cytotoxicity of soil after phytoremediation measured with mitochondrial dehydrogenase and lactate dehydrogenase activities was lower compared to the sterilized soil before phytoremediation.

### 3.5. Assessment of Soil Microbial Activity 

The soil microbial activity estimated with dehydrogenase and fluorescein diacetate hydrolase activities in non-vegetated soil was about 65 and 5 µg g^−1^ of soil per 24 h, respectively (Table 5). In vegetated soil with alfalfa (A), significant increases of dehydrogenase and fluorescein diacetate hydrolase activities (by 3 and 3.4 times, respectively) were observed compared to non-vegetated soil. For dehydrogenase activity, no significant differences were observed in vegetated soil with different conditions compared to vegetated soil with alfalfa (A). The higher fluorescein diacetate hydrolase activity (70 µg g^−1^) was observed in the soil vegetated in the A+Fm+RW1+Rh+µb condition, which corresponds to 4.1-fold higher than in the non-inoculated alfalfa condition (A). In addition, fluorescein diacetate hydrolase activity was also higher in A+Fm and Fm+RW1 A+Fm+RW1 conditions, by 3- and 3.2-fold compared to A condition, respectively.

### 3.6. Soil Microbial Community Structure

After the reads from different datasets were filtered for quality, the bacterial gene dataset allowed us to retrieve a total of 91,236 sequences (across the six samples) that were assigned to 1205 OTUs after subsampling to 5802 reads per sample. For the fungal data set, 43,194 sequences (across six samples) were obtained and assigned to 3296 OTUs after subsampling to 5542 reads per samples. In the archaeal dataset, we obtained a total of 223,218 sequences (across six samples) that were assigned to 11 OTUs after subsampling to 999 reads per sample (Table S2, Supplementary materials). The mean OTU number observed within non-vegetated soil were 481, 9, and 604 in bacterial, archaeal, and fungal communities, respectively. In the A+Fm+RW1+Rh+µb microcosms, mean OTU number increased significantly only for bacteria by 29% (Table 6). Bacterial richness (Chao1 estimator) was significantly higher for the vegetated soil compared to non-vegetated soil. The diversity (Shannon index) for bacterial, archaeal, and fungal communities were estimated within non-vegetated soil, at 7.1, 0.9, and 1.21, respectively. The bacterial and fungal diversity appeared to be influenced by the condition A+Fm+RW1+Rh+µb compared to non-vegetated one (Table 6). Moreover, the PCoA ordinations showed a grouping of bacteria, fungi, and archaeal community structure in response to the conditions non-vegetated and A+Fm+RW1+Rh+µb (Figure S2, Supplementary materials). 

**Figure 3:**
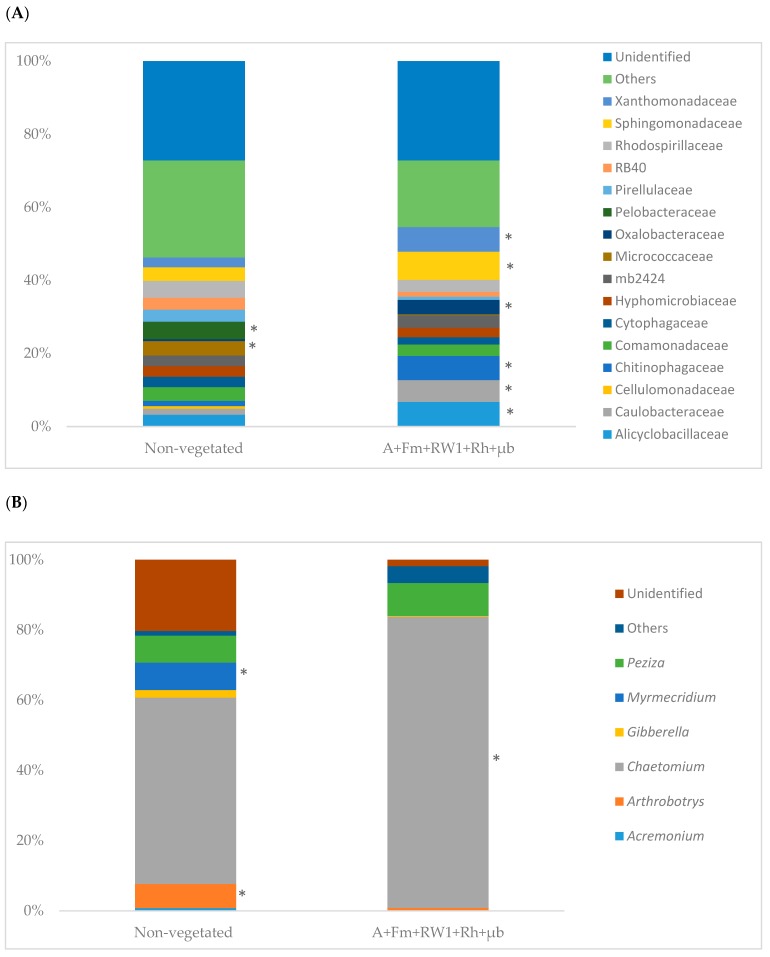
Relative abundances of major (**A**) bacterial Family of 16S rRNA gene dataset and (**B**) genera of fungal ITS dataset. (*) indicate significant differences between different bacterial family and fungal genus on non-vegetated soil and A+Fm+RW1+Rh+µb condition according to the Tukey (HSD) test (*p* ≤ 0.05).

After six months of culture, non-vegetated and A+Fm+RW1+Rh+µb conditions showed similarities in bacterial community composition at the family level. In the non-vegetated soil, the 13 most dominant members were found to be Alicyclobacillaceae, Bacillaceae, Comamonadaceae, Cytophagaceae, Hyphomicrobiaceae, mb2424, Micrococcaceae, Pelobacteraceae, Pirellulaceae, RB40, Rhodospirillaceae, Sphingomonadaceae, and Xanthomonadaceae with 2.5% to 6% (Figure 3A). While the abundance of Alicyclobacillaceae, Bacillaceae, Caulobacteraceae, Chitinophagaceae, Cytophagaceae, Sphingomonadaceae, and Xanthomonadaceae appeared to increase significantly in A+Fm+RW1+Rh+µb soil compared to non-vegetated one, the abundance of Micrococcaceae and Pelobacteraceae decreased. No differences of the abundances were detected for Comamonadaceae, Cytophagaceae, Hyphomicrobiaceae, mb2424, Pirellulaceae, RB40, and Rhodospirillaceae between the both conditions.

For the fungal community (Figure 3B), all samples showed similar composition at the genus level. The non-vegetated soil was dominated by members of the genus *Arthrobotrys*, *Chaetomium*, *Myrmecridium,* and *Peziza* with 6.9, 53, 7.8, and 7.7% respectively. The abundance of *Chaetomium* increased significantly in the A+Fm+RW1+Rh+µb condition by 36% compared to the non-vegetated soil. The abundance of *Arthrobotrys* and *Myrmecridium* decreased significantly in A+Fm+RW1+Rh+µb soil compared to non-vegetated one. No differences of the abundances were detected for *Peziza* between both conditions. 

## 4. Discussion

In order to investigate the potential of aided phytoremediation in cleaning-up aged PCDD/F contaminated soil, a microcosm experiment using alfalfa associated with several biological amendments (AMF inoculum, rhamnolipids, a dioxin-degrading bacteria *S. wittichii* RW1, and the native microbiota) was carried out. PCDD/F dissipation, the soil microbial community structure, and the toxicity of the contaminated soil were investigated. 

Firstly, our results showed that the addition of AMF inoculum, rhamnolipids, native microbiota, and *S. wittichii* RW1 to PCCD/F-aged contaminated soil has a positive effect on alfalfa aerial growth and chlorophyll content compared to alfalfa without amendment. These results were expected and are in agreement with several previous studies, which showed that AMF increase plant development in contaminated soils by improving mineral uptake of P, Mn, K, Cu, and Fe [24,41,42]. Thus, it has already been shown that the introduction of rhamnolipids in PAH-polluted soil increased the permeability of ryegrass root cells, which may also increase the nutrient uptake [43]. On the other hand, the production of indole-3-acetic acid by *Sphingomonas* sp. can also explain the enhancement of plant growth [44]; this compound also produced by plants is the most common plant hormone of auxin class, known to induce cell elongation and cell division [45]. The higher plant growth under the amended conditions can also be explained by a decrease in soil phytotoxicity. Indeed, a PCDD-induced toxicity was demonstrated in *Arabidopsis* by a decrease in seed germination, a loss in fresh weight and a striking decrease in chlorophyll content [14]. In the present study, the cytotoxicity of PCDD/F in soil, measured through mitochondrial dehydrogenase and lactate dehydrogenase activity assessments, was found to be significantly reduced after 24-week phytoremediation. Generally, the soil toxicity diminishes with the decrease of TEQ PCDD/F concentrations [46]. In the present work, the TEQ PCDD/F concentrations decreased by 5.47 TEQ ng kg^−1^ when soil was vegetated with alfalfa added with the various amendments (A+Fm+RW1+Rh+µb). These lower phytotoxicities and cytotoxicities could be explained by the decrease of highly toxic PCCD/F congeners in soil. Indeed, our results showed a total dioxins/furans dissipation of 23% (which corresponds to 48 ng.kg^−1^ of soil) after six months of culture in the vegetated soil combined with the four amendments compared to the non-vegetated soil. This dissipation could be assigned to the synergic effect of the supplemented amendments (AMF inoculum, rhamnolipids, the dioxin-degrading bacteria *S. wittichii* RW1, and the native microbiota). The adsorption of PCDD/F to the soil particles is favorable due to their hydrophobic properties and becomes stronger as the number of chlorine atoms increases; limiting the biodegradation process of highly substituted PCDD/F [47,48]. It is widely known that biosurfactants such as rhamnolipids can increase the water solubility of lipophilic contaminants such as polychlorinated biphenyls (PCBs) or polynuclear aromatic hydrocarbons from soil and therefore their bioavailability [49]. Several studies have demonstrated the ability of biosurfactants to increase the desorption of hydrophobic contaminants from soil organic matter and make them available for biodegradation [16,17,18]. Enhancement of phytoremediation efficiency in petroleum hydrocarbon-contaminated soils in the presence of rhamnolipids has already been described previously [25,50]. However, the application of surfactants to improve phytoremediation of contaminated soils with chlorinated compounds has received very little attention in the literature so far [51]. Manickam et al. [52] reported the highest solubilization rhamnolipid property, which enhanced biodegradation of hexachlorocyclohexane isomers by *Sphingomonas* sp. in artificially polluted soil after 30 days of culture. AMF played an important role in survival and in plant growth and subsequently in the composition as well as the quantity of root exudates [53,54]. Moreover, it was also demonstrated that mycorrhizal inoculation influenced bacterial, archaeal, and fungal community abundance and diversity in the soil [31]. In addition, it was also reported in previous studies that AMF could enhance chlorinated compound dissipation through increasing and selecting the number of degrading-microorganisms in the mycorrhizosphere [23,50]. In this work, the degrading-microbial consortia corresponded to the reintroduced native microbiota (µb) from the aged-contaminated soil and *S. wittichii* RW1, a bacteria of great interest in the bioremediation of polluted soils for its ability to metabolize a large number of toxic polychlorinated dioxins [55,56]. This strain uses dioxygenase-catalyzed angular ring hydroxylation to oxidize several chlorinated PCDD/F and to rapidly mineralize dibenzodioxin and dibenzofuran [57,58]. Some rhizosphere microorganisms probably act in tandem to effectively enhance the PCCD/F breakdown [59,60,61,62]. However, the degradation of highly chlorinated congeners is restricted to a few strains, such as *Sphingomonas wittichii* RW1, *Pseudomonas mendocina* NSYSU, and *Pseudallescheria boydii* [56,63,64]. On the other hand, our results pointed out a negligible amount of PCDD/F in alfalfa shoot dry weight in comparison with the amount removed (5.47 TEQ ng kg^−1^ corresponding to 48 ng kg^−1^ soil), suggesting that the absorption by plant contributed minimally to PCCD/F removal. Indeed, various studies showed that PCCD/F are not transported to plant roots and then not transferred into aboveground plant tissue because of their hydrophobicity [65]. Therefore, we presume that the removal PCDD/F for the condition A+Fm+RW1+Rh+µb results from the stimulation of soil microbial biodegradation activity. In fact, dehydrogenase and fluorescein diacetate hydrolase soil microbial activities were significantly higher in vegetated soil compared to non-vegetated soils. Similar results were also described by Tu et al. [66] who showed that alfalfa significantly increased both dehydrogenase and fluorescein diacetate hydrolase activities in polychlorinated biphenyl polluted soil compared to the non-vegetated soil after two-years remediation. The stimulation of these enzyme activities could be also explained by the increase of microbial diversity and richness. The mean of bacterial OTUs, richness (Chao 1 estimator), and diversity (Shannon index) increased significantly in Fm+RW1+Rh+µb condition compared to the control non-vegetated soil. A shift has also been observed for the fungal community, which corresponds to an increase of fungal diversity in Fm+RW1+Rh+µb condition. The bioremediation of PCDD/F-contaminated soil requires diverse microbial degradation processes involving the activity of specialized microbial communities. It was suggested that PCDD/F degradation began by an initial dechlorination process to free several chlorine-substituted positions downstream for the hydroxylation and cleavage of aromatic rings [67]. Reductive dehalogenation and oxidative degradation, mediated by specific anaerobic and aerobic microbial populations, respectively, have been proposed as mechanisms involved in the transformation of PCDD/F [68]. Therefore, to identify the potential microbial groups associated to PCDD/F degradation in our aged contaminated soil, the amplicon sequencing data were examined. So far, numerous bacterial at the family level Sphingomonadaceae and Bacillaceae-related populations have been associated with the degraders of PCDD/F and similar pollutants in contaminated soils [69,70]. Our findings showed that the abundance of Alicyclobacillaceae, Bacillaceae, Caulobacteraceae, Chitinophagaceae, Cytophagaceae, Sphingomonadaceae, and Xanthomonadaceae increased significantly in the Fm+RW1+Rh+µb condition compared to the non-vegetated one. These families could play a role in the PCDD/F degradation under our experimental conditions. Moreover, the ability of fungi to degrade PCDD/F has also been described. In saprotrophic fungi, several oxidative enzymes are involved in PCDD/F degradation such as cytochrome P-450, lignin peroxidase, manganese peroxidase, and laccase [71]. Our findings showed also that *Chaetomium* abundance was higher in the Fm+RW1+Rh+µb condition compared to non-vegetated soil. These fungal species are able to degrade lignin leading to various hydrophilic metabolites when grown in the presence of dibenzofuran [72,73]. Thus, taken together, our study suggested that alfalfa in association with the different biological amendments have quantitative and qualitative effects on shaping soil microbial community and could modify the PCDD/F dissipation processes consequently.

## 5. Conclusions

Our study demonstrated that phytoremediation of aged PCCD/F-contaminated soil was improved using the combined effect of alfalfa, AMF inoculum, rhamnolipids amendment, introduction PCDD/F degrading-bacteria *S. wittichii* RW1, and reintroduction of native microbiota, which probably possess the genetic resources associated with the degradation of PCDD/F. In the combined condition, PCDD/F dissipation was concomitant with a significant cytotoxicity reduction of the contaminated soil and higher microbial enzyme activities compared to the non-vegetated soil. Furthermore, this work allowed us to investigate the microbiome of PCDD/F-contaminated soil and to identify several bacterial (Bacillaceae, Sphingomonadaceae) and fungal (*Chaetomium*) groups known to be involved in PCDD/F degradation. The combined use of these various amendments may have some potential such as a biotechnological approach in cleaning up PCDD/F-aged polluted soils and in reducing the toxicity risk of such polluted soils. 

## Figures and Tables

**Table 1:** Physico-chemical parameters of the aged polluted sterilized soils.

Parameters	Values
Clay (g/kg)	135 ± 10
Silt: Fine particle (g/kg)	149 ± 9
Silt: Coarse particle (g/kg)	431 ± 13
Sand: Fine particle (g/kg)	257 ± 16
Sand: Coarse particle (g/kg)	28 ± 6
Organic C (g/kg)	25.1 ± 6
Total N (g/kg)	1.53 ± 0.5
Organic matter (g/kg)	43.4 ± 8
Total CaCO_3_ (g/kg)	<1
P_2_O_5_ (g/kg)	0.027 ± 0.027
Mn (cmol+/kg)	0.135 ± 0.008
Ca (g/100g)	0.565 ± 0.056
Na (g/100g)	0.697 ± 0.098
Al (cmol+/kg)	0.0469 ± 0.0293
Fe (cmol+/kg)	0.0089 ± 0.0013
Si (g/100g)	0.207 ± 0.065
Mg (cmol+/kg)	1.06 ± 0.3
pH	6.34 ± 0.035
PCDD/F (ng/kg)	208 ± 40

**Table 2:** Experimental design: Description of various conditions tested. x symbol means presence of the treatment in the conditions.

Conditions	Control	Alfalfa (A)	*F. mosseae* (Fm)	*S. wittichii* (RW1)	Rhamnolipids (Rh)	Native Microbiota (µb)
Non-vegetated	x					
A		x				
A+Fm		x	x			
A+Fm+RW1		x	x	x		
A+Fm+RW1+Rh		x	x	x	x	
A+Fm+RW1+Rh+µb		x	x	x	x	x

**Table 3:** Total, arbuscular, and vesicular mycorrhizal colonization of alfalfa in response to different conditions. Conditions consisted of non-inoculated alfalfa (A), combined with *F. mosseae* (A+Fm), plus *S. wittichii* RW1 (A+Fm+RW1), plus rhamnolipids Rh (A+Fm+RW1+Rh), plus microbiota µb (A+Fm+RW1+Rh+µb). Shoots and roots dry weight of alfalfa (A), combined with *F. mosseae* (A+Fm), plus *S. wittichii* RW1 (A+Fm+RW1), plus rhamnolipids Rh (A+Fm+RW1+Rh), plus microbiota µb (A+Fm+RW1+Rh+µb) grown in microcosms for six months on the aged polluted soil. Different letters indicate significant differences between conditions according to the ANOVA one-way test (*p* ≤ 0.05). Values are means ± s.d. (*n* = 5).

		A	A+Fm	A+Fm+RW1	A+Fm+RW1+Rh	A+Fm+RW1+Rh+µb
Mycorrhizal rate (%)	Total	0	47 ± 2 a	39 ± 4 ab	30 ± 8 b	40 ± 6 ab
	Arbuscular	0	12 ± 2 b	7 ± 1 b	7 ± 3 ab	10 ± 1 b
	Vesicular	0	31 ± 3 a	25 ± 8 ab	20 ± 6 b	23 ± 2 ab
Plant dry weight (mg plant^−1^)	Shoot dry weight	97 ± 1 a	279 ± 1.6 d	171 ± 30 b	191 ± 15 b	207 ± 18 bc
	Root dry weight	60 ± 12 ab	67 ± 19 b	76 ± 3 bc	39 ± 8 a	55 ± 2 ab

**Table 4:** Lactate dehydrogenase (LDH) activity and mitochondrial (MDH) activity after lung and liver cells’ exposure to organic extracts of contaminated soil planted with alfalfa and combined with Fm, RW1, Rh, and µb compared to soil samples before phytoremediation. *p* values in bold indicate significant differences between A+Fm+RW1+Rh+µb condition and control soil before phytoremediation according to the Mann–Whitney test (*p* ≤ 0.05). Values are means + s.d. (*n* = 12).

Cells	Enzymes	A+Fm+RW1+Rh+µb (%)
**Lung Cells (Beas2B)**	LDH activity vs. control	−44 ± 24 (*p* < 0.0001)
	MDH activity vs. control	+5 ± 1 (*p* = 0.751)
**Liver Cells (HepG2)**	LDH activity vs. control	−5 ± 1 (*p* = 0.0001)
	MDH activity vs. control	+18 ± 9 (*p* < 0.0001)

**Table 5:** Dehydrogenase and Fluorescein diacetate hydrolase activities in polluted soil of different conditions. Conditions consisted of non-inoculated alfalfa (A), alfalfa combined with *F. mosseae* (A+Fm), plus *S. wittichii* RW1 (A+Fm+RW1), plus rhamnolipids Rh (A+Fm+RW1+Rh), plus microbiota µb (A+Fm+RW1+Rh+µb) grown in microcosms for six months. Different letters indicate significant differences between conditions according to the Kruskal–Wallis test (*p* ≤ 0.05). Values are means ± s.d. (*n* = 4).

Conditions	Dehydrogenase (µg g^−1^ of Soil/24 h)	Fluorescein Diacetate Hydrolase (µg g^−1^ of Soil)
Non-vegetated	65 ± 3 a	5.0 ± 0.1 a
A	196 ± 28 bcd	17.0 ± 6.0 b
A+Fm	288 ± 94 cd	51.0 ± 10.0 c
A+Fm+RW1	186 ± 36 bc	55.0 ± 7.0 c
A+Fm+RW1+Rh	173 ± 22 b	25.0 ± 4.0 b
A+Fm+RW1+Rh+µb	281 ± 31 d	70.0 ± 7.0 d

**Table 6:** Alpha diversity metrics for rhizosphere samples collected from non-planted polluted soil and in inoculated alfalfa with Fm plus RW1 plus Rh plus µb (A+Fm+RW1+Rh+µb) grown in microcosms for six months. Values represent mean ± SD. Different letters indicate significant differences between A+Fm+RW1+Rh+µb and non-planted soil according to Tukey (HSD) test on observed OTUs, Chao1 richness estimate, and Shannon diversity indices on the community structures of Bacteria, Archaea, and Fungi. Values are significant at *p* < 0.05.

		Observed OTUs	Chao1 Richness Estimate	Shannon Diversity Index
**Bacteria**	Non-vegetated	481 ± 23 a	488 ± 34 a	7.10 ± 0.14 a
	A+Fm+RW1+Rh+µb	618 ± 57 b	657 ± 51b	7.50 ± 0.13 b
**Archaea**	Non-vegetated	9 ± 1 a	8 ± 1a	0.90 ± 0.42 a
	A+Fm+RW1+Rh+µb	7 ± 2 a	7 ± 1 a	1.30 ± 0.05 a
**Fungi**	Non-vegetated	604 ± 180 a	3890 ± 406 a	1.21 ± 0.80 a
	A+Fm+RW1+Rh+µb	644 ± 84 a	4108 ± 378 a	2.44± 0.26 b

## Supplementary Materials

The following are available online at https://www.mdpi.com/2076-2607/7/11/523/s1, Figure S1: Repartition (%) of PCDD/F in the A+Fm+RW1+Rh+µb condition after 6 months of culture in microcosms, Figure S2: Principal coordinates analysis (PCoA) of (a) Bacteria, (b) Fungi, and (c) Total Archaeal community. Red color for the control non-vegetated soil, blue color for A+Fm+RW1+Rh+µb condition, Table S1: Summary of sequencing bioinformatics identification of OUT, Table S2: Description of the performed tests.
