# Supplementary material for: Aided Phytoremediation to Clean Up Dioxins/Furans-Aged Contaminated Soil: Correlation between Microbial Communities and Pollutant Dissipation
Source: Microorganisms. 2019 Nov 3;7(11):523. doi: 10.3390/microorganisms7110523 (PMC6920798; doi:10.3390/microorganisms7110523)
Supplement: Supplementary file 1 [file microorganisms-07-00523-s001.pdf]

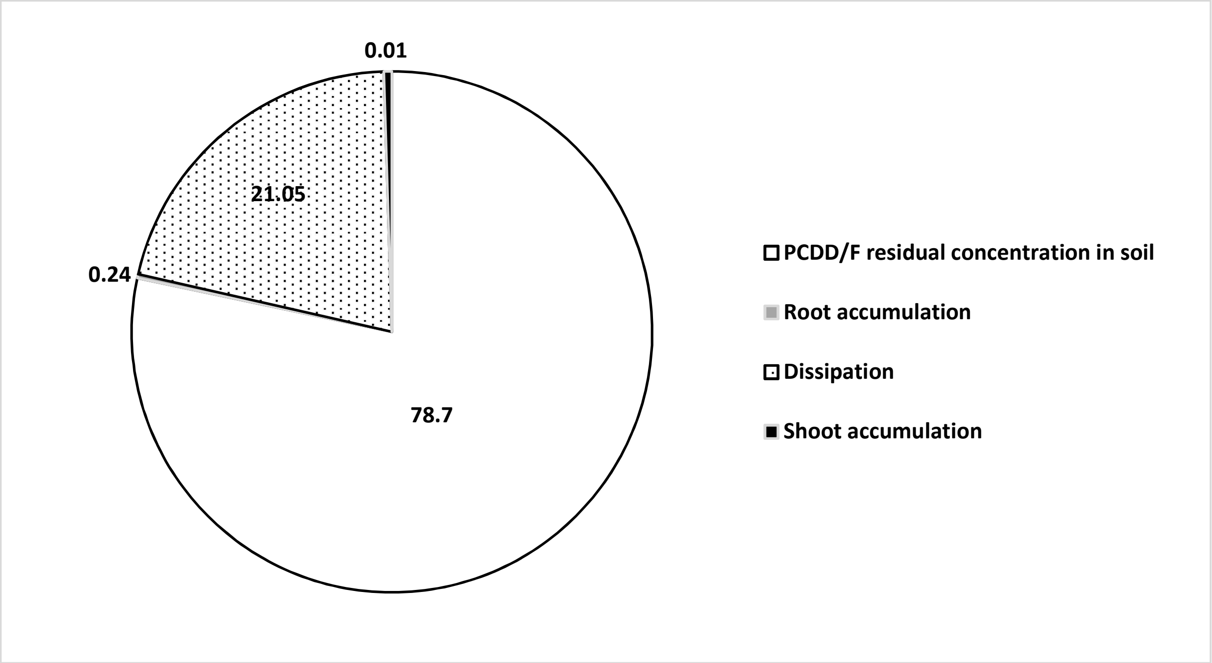

Figure S1. Repartition (%) of PCDD/F in the A+Fm+RW1+Rh+µb condition after six months of culture in microcosms.

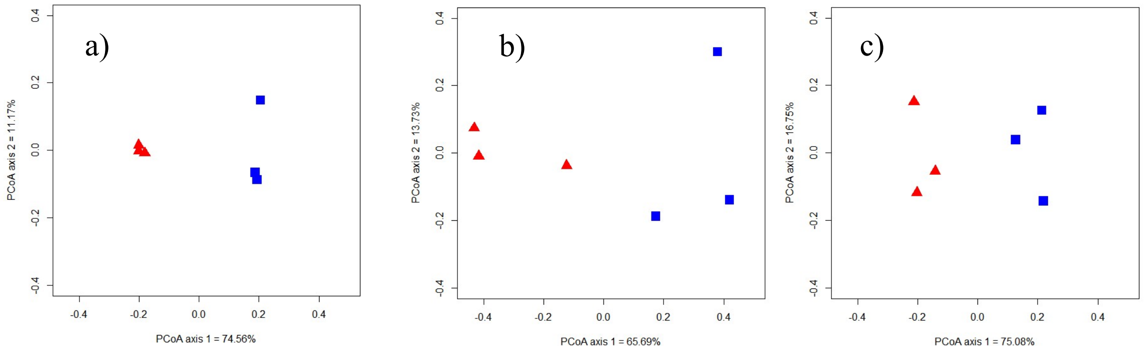

Figure S2. Principal coordinates analysis (PCoA) of (a) Bacteria, (b) Fungi and (c) Total Archaeal community. Red color for the control non-vegetated soil, blue color for A+Fm+RW1+Rh+µb condition

Table S1. Summary of sequencing bioinformatics identification of OTU.

	Bacteria	Fungi	Archaea
No sequences (sum)	91,236	43,194	223,218
No seq. (mean)	15,206	7,199	37,203
No seq. filtred	5,505	2,354	12,254
No seq. chimeras (mean)	991	725	1,023
No seq. singleton (mean)	2,664	1,883	13,770
No samples	6	6	6
No seq. saved (mean)	6,046	2,962	13,216
OTUs (sum)	1,205	3,296	11

Table S2: Description of the performed tests

Methods kind	Name	Descriptions
Physicochemical	- AMF root colonization<br>- Root and shoot dry weights<br>- Pigment contents<br>- Dioxins/furans content<br>- DNA quantification	- Microscopic<br>- Balance<br>- Spectrophotometer<br>- GC/HRMS<br>-
Biochemical	- Soil enzyme assays (Dehydrogenase and Fluorescein diacetate hydrolase)<br>- DNA extraction	- Spectrophotometer and spectrofluorometer<br>- Macherey-Nagel kit
Biological	- Cytotoxicity tests<br>- PCR and amplicon sequencing	- Cytotoxicity Detection Kit lactate dehydrogenase and Cell Proliferation Reagent WST-1<br>- Illumina MiSeq
Bioinformatic	- QIIME<br>- Mothur	- Brazilian Microbiome Project
Statistical	- Shapiro and Bartlett tests<br>- ANOVA one-way,<br>- Mann-Whitney<br>- Kruskal-Wallis tests	
